# Association between Vitamin D and Heart Failure Mortality in 10,974 Hospitalized Individuals

**DOI:** 10.3390/nu13020335

**Published:** 2021-01-23

**Authors:** Kenya Kusunose, Yuichiro Okushi, Yoshihiro Okayama, Robert Zheng, Miho Abe, Michikazu Nakai, Yoko Sumita, Takayuki Ise, Takeshi Tobiume, Koji Yamaguchi, Shusuke Yagi, Daiju Fukuda, Hirotsugu Yamada, Takeshi Soeki, Tetsuzo Wakatsuki, Masataka Sata

**Affiliations:** 1 Department of Cardiovascular Medicine, Tokushima University Hospital, Tokushima 770-8503, Japan; yuuitirou_0110@yahoo.co.jp (Y.O.); pangtong2004@yahoo.ne.jp (R.Z.); myasan.abe@gmail.com (M.A.); isetaka@tokushima-u.ac.jp (T.I.); tobiume.takeshi@tokushima-u.ac.jp (T.T.); yamakoji3@tokushima-u.ac.jp (K.Y.); syagi@tokushima-u.ac.jp (S.Y.); daiju.fukuda@tokushima-u.ac.jp (D.F.); soeki@tokushima-u.ac.jp (T.S.); wakatsukitz@tokushima-u.ac.jp (T.W.); masataka.sata@tokushima-u.ac.jp (M.S.); 2 Clinical Research Center for Developmental Therapeutics, Tokushima University Hospital, Tokushima 770-8503, Japan; y-okayama@tokushima-u.ac.jp; 3 Center for Cerebral and Cardiovascular Disease Information, National Cerebral and Cardiovascular Center, Osaka 564-8565, Japan; nakai.michikazu@ncvc.go.jp (M.N.); ysumi@ncvc.go.jp (Y.S.); 4 Department of Community Medicine for Cardiology, Tokushima University Graduate School of Biomedical Sciences, Tokushima 770-8503, Japan; yamadah@tokushima-u.ac.jp

**Keywords:** heart failure, vitamin D, mortality, big data

## Abstract

A broad range of chronic conditions, including heart failure (HF), have been associated with vitamin D deficiency. Existing clinical trials involving vitamin D supplementation in chronic HF patients have been inconclusive. We sought to evaluate the outcomes of patients with vitamin D supplementation, compared with a matched cohort using real-world big data of HF hospitalization. This study was based on the Diagnosis Procedure Combination database in the Japanese Registry of All Cardiac and Vascular Datasets (JROAD-DPC). After exclusion criteria, we identified 93,692 patients who were first hospitalized with HF between April 2012 and March 2017 (mean age was 79 ± 12 years, and 52.2% were male). Propensity score (PS) was estimated with logistic regression model, with vitamin D supplementation as the dependent variable and clinically relevant covariates. On PS-matched analysis with 10,974 patients, patients with vitamin D supplementation had lower total in-hospital mortality (6.5 vs. 9.4%, odds ratio: 0.67, *p* < 0.001) and in-hospital mortality within 7 days and 30 days (0.9 vs. 2.5%, OR, 0.34, and 3.8 vs. 6.5%, OR: 0.56, both *p* < 0.001). In the sub-group analysis, mortalities in patients with age < 75, diabetes, dyslipidemia, atrial arrhythmia, cancer, renin-angiotensin system blocker, and β-blocker were not affected by vitamin D supplementation. Patients with vitamin D supplementation had a lower in-hospital mortality for HF than patients without vitamin D supplementation in the propensity matched cohort. The identification of specific clinical characteristics in patients benefitting from vitamin D may be useful for determining targets of future randomized control trials.

## 1. Introduction

The main treatment medications for heart failure (HF) remains to be β-blockers, angiotensin converting enzyme inhibitors, angiotensin receptor blockers, and aldosterone receptor antagonists in the guidelines [1]. Although it is well known that these medications can reduce the incidence of adverse cardiac events and improve cardiac function, HF is still a main cause of death worldwide [2]. Thus, supplementary treatment methods continue to be explored for improving the outcome of HF.

Vitamin D is a steroid hormone belonging to a group of lipid-soluble vitamins. Recently, many papers showed that a broad range of chronic conditions have been associated with vitamin D deficiency [3,4,5]. Around 90% of chronic HF patients have insufficient vitamin D levels, even in sunny climates [6,7]. Vitamin D has pleiotropic effects in the pathology of chronic HF [8]. Despite current evidence regarding the association of vitamin D with HF, there are many controversial results in previous clinical trials [9,10,11]. In these trials, lack of a large sample size and the small number of high-risk patients are major limitations. Our hypothesis was that vitamin D supplementation was associated with a decreased risk of in-hospital death in HF patients with specific clinical characteristics. Therefore, we sought to evaluate the outcomes of patients with vitamin D supplementation compared with a matched cohort using real-world big data based on HF hospitalizations.

## 2. Materials and Methods

### 2.1. Study Population

**Figure 1:**
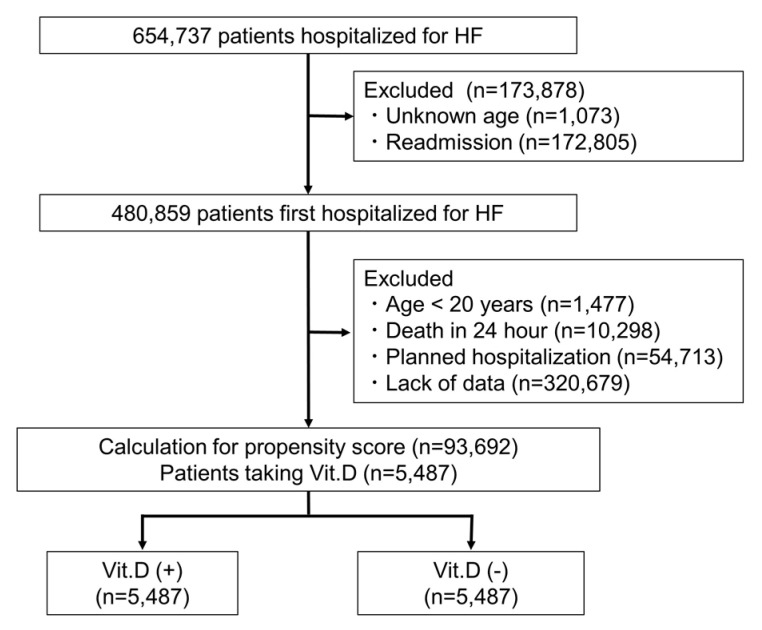
Flowchart of this study. HF, heart failure; Vit D, vitamin D supplementation.

The study population was composed of hospitalized patients between April 2012 and March 2017 in The Japanese Registry of All Cardiac and Vascular Diseases and the Diagnosis Procedure Combination (JROAD-DPC) database. JROAD-DPC is a nationwide registry, a medical database with information of admission and discharge for cardiovascular diseases, clinical examinations and treatment status, patient status and hospital overview. JROAD-DPC database integrates the information composed by JROAD-DPC data, with analysis data sets covering 5.1 million cases in 1022 hospitals between April 2012 and March 2017. The identification of HF (I50.0, I50.1, I50.9) hospitalization was based on the International Classification of Diseases (ICD)-10 diagnosis codes. Data regarding patient age and sex, main diagnosis, comorbidity at admission, length of hospitalization and treatment content were extracted from the database. We recruited 654,737 patients hospitalized with HF. Diagnosis of HF was defined as the main diagnosis, admission-precipitating diagnosis or most resource-consuming diagnosis. We excluded patients of unknown age (*n* = 1073), readmission cases (*n* = 172,805), age < 20 years (*n* = 1477), death in 24 h after admission (*n* = 10,298), planned hospitalization (*n* = 54,713), and incomplete data (*n* = 320,679). As a result, total 93,692 (88,205 patients without vitamin D and 5487 patients with vitamin D) were recruited to assess hospital mortality (Figure 1). For vitamin D supplementation, oral 25(OH)D 3 (Calcifediol, Dedrogyl^®^) was prescribed at a daily dose of 0.5–1.0 μg/day based on the attending physician’s discretion.

### 2.2. Clinical Outcomes

The main outcome was in-hospital mortality (total number of deaths during hospitalization). Death ≤ 7 and 30 days after admission was assessed as secondary outcomes.

### 2.3. Sample Matching

Propensity score (PS) matching was used to reduce confounding effects related to differences in patient background. PS was estimated with a logistic regression model, with vitamin D supplementation as the dependent variable and the following clinically relevant covariates; age, sex, body mass index (BMI), smoking, New York Heart Association functional classification (NYHA), comorbidities (hypertension: HT, diabetes: DM, dyslipidemia: DL, osteoporosis, atrial fibrillation/atrial flutter: Af/AFL, stroke, myocardial infarction: MI, peripheral vascular disease: PVD, renal disease, liver failure, chronic obstructive pulmonary disease: COPD, rheumatoid arthritis: RA, dementia, cancer), treatment (catecholamine, intra-aortic balloon pumping: IABP, percutaneous cardiopulmonary support: PCPS, ventilation, hemodialysis: HD, percutaneous coronary intervention: PCI). These covariates were chosen for their potential association with reference to risk factor of heart failure and in-hospital mortality [12,13,14]. Matching was performed with greedy-matching algorithm (ratio = 1:1 without replacement), with a caliper of width 0.2 standard deviations of the logistic of the estimated propensity score. After matching, vitamin D and non-vitamin D groups of 5487 patients each were included in the final analysis. The area under the curve was 0.785 and the consistency of PS densities was matched after matching (Supplemental Figure S1). The balance of each covariate before and after matching between the 2 groups was evaluated by standardized differences. Absolute value of standardized differences less than 10% was considered to be a relatively small imbalance. Because the propensity score included cases in which vitamin D was used under the insurance of Japan (renal disease, osteoporosis, and dialysis), we believed the propensity score accounted for the factors that influence the prescription of vitamin D by general physicians in this analysis.

### 2.4. Statistical Analysis

Continuous variables are expressed as mean ± SD for parameters with normal distribution, as median (interquartile range; IQR) for parameters with skewed distribution, and categorical variables as proportion (%). We checked characteristics between groups with and without vitamin D supplementation using standardized difference. After matching, we estimated the OR for in-hospital mortality (total, within 7 days, 30 days) using mixed-effects logistic regression model with each institute as a random effect. We also analyzed subgroups in the PS-matched cohort. In-hospital mortality was assessed using Kaplan–Meier curves and log-rank test to compare the two groups. To clarify the beneficial group of vitamin D supplementation, odds ratios (ORs) and their 95% confidence interval (CI) for in-hospital mortality were calculated using multivariate models of multinomial logistic regression analysis in vitamin D (+) and vitamin D (−) groups. All statistical tests were 2-sided and *p* values less than 0.05 were considered statistically significant. Statistical analysis was performed using SAS version 9.4 and JMP version 14.0.

## 3. Results

### 3.1. Patient Characteristics

A total of 52.2% of patients in this study were male. Mean age was 79 ± 12 years, and half of all patients had hypertension (52.9%). Over 60% of the patients were NYHA class III or IV. Patients with vitamin D supplementation were more likely to have a history of chronic kidney disease, osteoporosis, hypoparathyroidism, or hemodialysis. There are differences for age, gender, BMI, smoking, hypertrophic cardiomyopathy, atrial fibrillation/atrial flutter, and rheumatoid arthritis between two groups. Around 19.7% took angiotensin converting enzyme inhibitors (ACE-I) or angiotensin-receptor blocker (ARB) and 9.1% took beta-blockers. About 19.4% of the patients took loop diuretic and 10.1% took K-sparing diuretics.

After propensity score matching, 10,974 patients were included in the survival analysis. In the matched cohort, there were no significant differences between groups for age, gender, comorbidities, and treatments (Table 1).

### 3.2. Outcomes

In-hospital mortality, mortality within 7 days and within 30 days of hospitalization are summarized in Table 2. Even after matching, patients with vitamin D supplementation had significantly lower in-hospital mortality (6.5 vs. 9.4%, *p* < 0.001; OR, 0.67, 95% CI: 0.58–0.77), mortality within 7 days of hospitalization (0.9 vs. 2.5%, *p* < 0.001; OR, 0.34, 95% CI: 0.25–0.48), and mortality within 30 days of hospitalization (3.8 vs. 6.5%, *p* < 0.001; OR, 0.56, 95% CI: 0.47–0.67).

**Figure 2:**
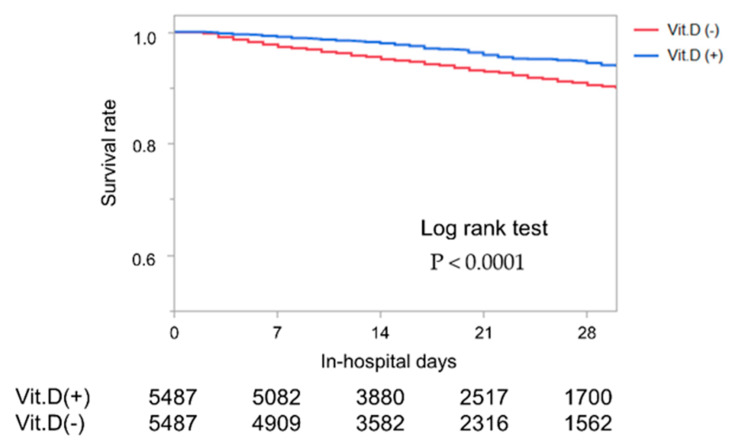
Kaplan Meier curves of in-hospital mortality and hospitalization days. Comparison between with and without vitamin D (Vit.D) supplementation.

Kaplan–Meier curves of in-hospital mortality were shown in Figure 2. Vitamin D supplementation was strongly associated with survival rate (*p* < 0.001).

Multivariate analysis was performed with covariates that were significant in the univariate analysis to assess the association with in-hospital mortality for all patients. Major contributors were age, BMI, NYHA, hypertension, peripheral vascular disease, chronic kidney disease, artificial ventilation, PCI, catecholamine, and atrial fibrillation/flutter in this cohort. After adjustment of clinical backgrounds, vitamin D supplementation was associated with low in-hospital mortality (OR, 0.63, 95% CI: 0.49–0.81, *p* < 0.001) (Table 3A).

In multivariate analysis, there were many same risks (Table 3B,C: age, hypertension, artificial ventilator, PCI, catecholamine) for in-hospital mortality in vitamin D (+) and vitamin D (−). We have checked the difference of ORs between two groups for the risk distribution. Osteoporosis patients seemed to be protected in vitamin D (+) group, however, to be at increased risk in vitamin D (−) group. Based on this result, especially osteoporosis patients may have benefits from vitamin D supplementation during heart failure admissions. For hemodialysis and chronic kidney disease, there seemed to be small benefit in using vitamin D from ORs. BMI was associated with death in patients taking vitamin D (OR, 0.91, 95% CI: 0.87–0.95, *p* < 0.001), however, BMI was not associated with mortality in patients with vitamin D supplementation (OR, 0.97, 95% CI: 0.92–1.02, *p* = 0.22). The BMI may be an extra risk beyond the selection of patients with kidney disease or osteoporosis.

Predictive values using ROC analysis for in-hospital morality were good (Supplemental Figure S2: C-statistics: 0.85 for vitamin D (+) and 0.84 for vitamin D (−)) compared with the previous prediction models [15]. Thus, we thought that risk prediction performance was not different in both populations.

### 3.3. Subgroup-Analysis

**Figure 3:**
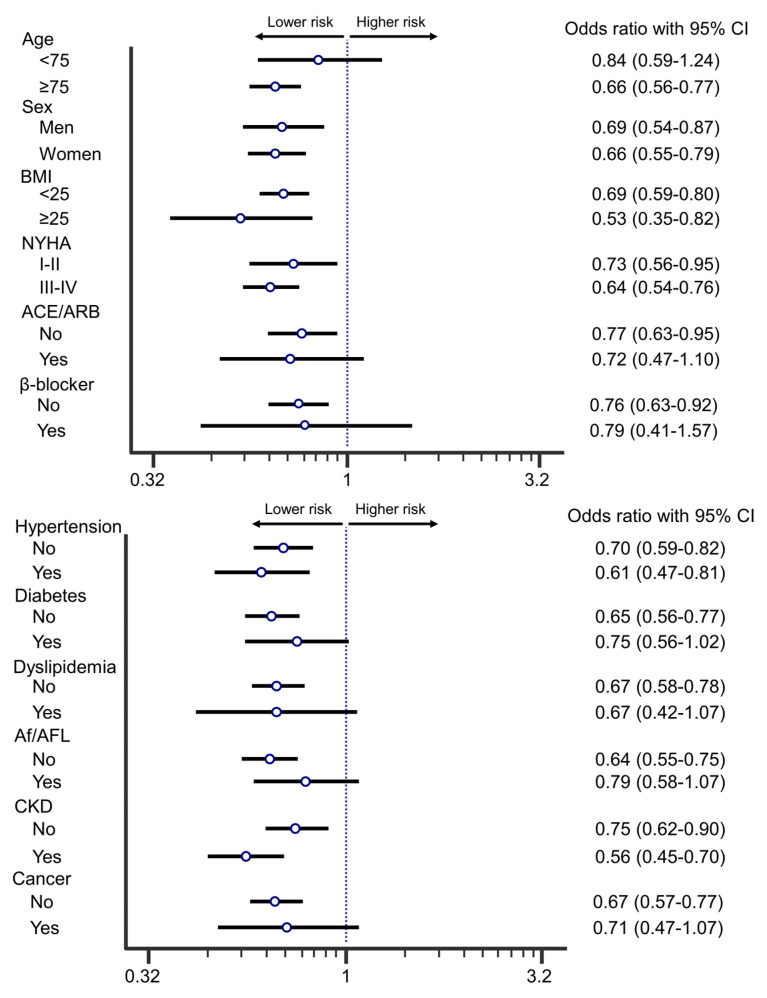
Odds ratio of in-hospital mortality. Patients with vitamin D compared with matched patients without vitamin D. Dots and lines mean OR and 95% CI, respectively.

Mortality in each sub-group, forest plots of OR are shown in Figure 3. Regardless of gender, BMI, NYHA, hypertension, and chronic kidney disease, patients with vitamin D supplementation had significantly lower in-hospital mortality than matched patients. Mortalities in patients with age < 75 (OR, 0.84, 95% CI: 0.59–1.24, *p* = 0.54), diabetes (OR, 0.75, 95% CI: 0.56–1.02, *p* =0.06), dyslipidemia (OR, 0.67, 95% CI: 0.42–1.07, *p* = 0.09), Af/AFL (OR, 0.79, 95% CI: 0.58–1.07, *p* = 0.13), cancer (OR, 0.71, 95% CI: 0.47–1.07, *p* = 0.10), ACEi/ARB medication (OR, 0.72, 95% CI: 0.47–1.10, *p* = 0.13), and β-blocker usage (OR, 0.80, 95% CI: 0.41–1.57, *p* = 0.51) were not affected by vitamin D supplementation. Thus, this analysis suggested that there were specific clinical characteristics in patients benefitting from vitamin D supplementation.

## 4. Discussion

The main findings of the present study were (1) HF patients with vitamin D supplementation had significantly lower in-hospital mortality and mortality within 7 and 30 days of hospitalization in the propensity matched cohort; (2) mortalities in patients with age < 75, diabetes, dyslipidemia, atrial arrhythmia, cancer, renin-angiotensin system blocker medication, and β-blocker were not affected by vitamin D supplementation; (3) by multivariate analysis we identified that it was mainly osteoporosis patients that benefit from being treated with vitamin D supplementation when they were admitted for HF. Mortality was consistently low in patients with vitamin D supplementation at 7 days, 30 days, and during hospitalization. On the other hand, there are specific clinical characteristics in HF patients who do not benefit much from vitamin D. The identification of specific clinical characteristics in patients benefitting from vitamin D may be useful in determining targets of future studies.

### 4.1. Impact of Vitamin D on HF Mortality

Although there is much evidence showing that a lack of vitamin D could result in poor prognosis among patients with HF, different studies have reported controversial results about the benefit of vitamin D supplementation in patients with HF. In recent years, there were some randomized control trials for the effects of vitamin D on patients with HF. For example, the Vitamin D treating patients with chronic heart failure (VINDICATE) study showed that vitamin D supplementation has beneficial effect on left ventricular (LV) structure and function [11]. An individual participant data meta-analysis observed an association between low vitamin D level and increased risk of all-cause mortality [16]. On the other hand, another meta-analysis reported that vitamin D supplementation did not improve LV ejection fraction and 6-min walk distance in the treatment of chronic HF [17]. A recent updated meta-analysis also reported that vitamin D supplementation was not significantly associated with reduced major adverse cardiovascular events [18].

While randomized clinical trials (RCT) provide a foundation for clinical evidence, trials are often performed in highly controlled environments with narrow inclusion and exclusion criteria, which reduces their generalizability and external validity. Highly protocolled care in an RCT may differ substantially from interventions in routine settings [19]. The Mendelian Randomization study is a new concept of analysis, however, the genetic variants are unclear in the vitamin D3 levels [20]. A notably limitation of these trials is that none were focused on vitamin D supplementation in patients with high-risk cohort including NYHA 3 and 4. From our subgroup analysis, patients, the effect of vitamin D on in-hospital mortality was seemed to be greater in NYHA III-IV patients compared with NYHA I-II (NYHA III-IV: OR: 0.63, *p* < 0.001 and NYHA I-II: OR: 0.72, *p* = 0.014). We believe that the key to proving the worth of vitamin D supplementation is to create clinical studies that also involve a significant number of decompensated HF patients.

### 4.2. Mechanisms of Vitamin D for HF

There are some theories for the association between vitamin D and HF prognosis. In HF, cardiac contraction and relaxation are affected due to overload of Ca2+ ions in myocardial cells. Lack of vitamin D may intervene with the functions of Ca2+ in myocardial cells, resulting in cardiomyocyte hypertrophy, intra-organisational inflammatory reaction and fibrosis [21,22]. Low vitamin D levels may activate the renin–angiotensin system [23], give rise to inflammatory reactions [24] and result in endothelial dysfunction [25]. Interestingly, our subgroup analysis suggested that patients without ACEi/ARB had received more beneficial effects from vitamin D in regards to in-hospital mortality. The effect of vitamin D was more pronounced in patients without ACEi/ARB usage, hence suggesting an activated renin–angiotensin system in these patients.

The effects of vitamin D on the cardiovascular system are additionally mediated through elevated parathyroid hormone levels [26]. An age-related increase in parathyroid hormone levels has been demonstrated in several studies [27]. In our cohort, elderly patients (with suspected elevation of parathyroid hormone) with vitamin D supplementation were associated with lower in-hospital mortality (age < 75: OR: 0.84, *p* = 0.40 and age ≥ 75: OR: 0.66, *p* < 0.001). This result may suggest a link between vitamin D and elevated parathyroid hormone levels in the cardiovascular system. Based on the basic knowledge of these mechanisms, the link between vitamin D and prognosis in HF may be explained.

### 4.3. Clinical Implication

Even with the current wealth of guidelines and recommendations about HF and development of many new treatment methods, HF is associated with a high in-hospital mortality [1]. For patients with HF, vitamin D supplementation is a low-cost low-risk choice, and certain patients may benefit greatly from this therapy. According to our data from the large high-risk HF cohort, patients with vitamin D supplementation had lower mortality, and specific clinical characteristics were linked to better in-hospital mortality. The identified specific clinical characteristics that might be useful for future RCT studies.

### 4.4. Limitations

The study based on ICD codes has several limitations. First, we analyzed only patients with HF hospitalized in facilities contributing to the database, which may lead to selection bias. Second, the database has no information on echocardiography or laboratory data to assess the prognosis of HF. Third, the database lacked information on the specific doses of vitamin D supplementation in each patient. Dose dependency was unable to be examined. Forth, propensity score-matching reports the potential differences between groups, with only a certain degree of accuracy. Despite the application of propensity matching to the comparator group of patients, this non-randomized observational study could still be subject to hidden biases related to patient selection, because of unknown unadjusted differences. To overcome this issue, we used treatment devices and catecholamine medication as markers of HF severity. All-cause mortality was used as the primary end point in our patient population. The most likely cause of death in our patient population is HF, given the known high-risk nature of our patient population. The patients in this study are mostly Japanese. Results may differ due to racial or cultural differences in other countries. The JROAD-DPC dataset extracts only a record which contains all types of cardiovascular diseases in any categories of diagnosis based on the DPC dataset in Ministry of Health, Labor and Welfare in Japan. The DPC dataset has already been validated in past studies [28]. However, we were unable to check the undefined diseases by the coding system in our final dataset. This registry data does not include laboratory data. However, there would be no difference in background between the two groups as we corrected for many confounding factors. Finally, the results cannot be applied to all heart failure admissions. The results can be applied to the group of patients who should receive vitamin D supplementation but did not get it. The reason is that there were many osteoporosis and hemodialysis patients in both groups. Thus, the vitamin D group was suspected to have higher serum 25(OH)D compared with the non-vitamin D group. Considering these limitations, the present study should be considered as a hypothesis generating study for future RCT studies.

## 5. Conclusions

Patients with vitamin D supplementation had a lower in-hospital mortality for HF than patients without vitamin D supplementation in this propensity matched cohort. The causality should be tested in the future RCTs in specific population based on our study.

## Figures and Tables

**Table 1:** Baseline characteristics before and after propensity score matching.

		Non Matching				Matching		
		All	Vit.D (+)	Vit.D (−)	std.diff (%)	Vit.D (+)	Vit.D (−)	std.diff (%)
Number		(*n* = 93,692)	(*n* = 5487)	(*n* = 88,205)		(*n* = 5487)	(*n* = 5487)	
Age average(years)		79 ± 12	80 ± 11	79 ± 13	10.6	80 ± 11	81 ± 11	8.4
Age(%)	20–30	0.2	0.1	0.2	−1.7	0.1	0.1	2.7
30–40		0.8	0.4	0.8	−6.2	0.4	0.4	−0.2
40–50		2.4	1.4	2.4	−7.8	1.4	1.1	2.5
50–60		4.8	3.6	4.8	−6.3	3.6	3.1	2.6
60–70		12.1	10.8	12.2	−4.4	10.8	9.8	3.3
70–80		23.0	23.8	22.9	2.0	23.8	22.1	3.9
80–90		39.8	42.4	39.7	5.5	42.4	44.2	−3.8
>90		17.0	17.6	17.0	1.7	17.6	19.3	−4.2
Male (%)		52.2	33.9	53.4	−40.1	33.9	31.4	5.3
BMI		22.7 ± 5.0	21.8 ± 4.1	22.7 ± 5.0	−20.8	21.8 ± 4.1	21.7 ± 4.1	1.9
Smoking		30.2	21.1	30.8	−22.1	21.1	18.9	5.7
NYHA	1	12.2	12.8	12.2	1.9	12.8	13.2	−1.0
2		24.4	24.5	24.3	0.3	24.5	25.0	−1.2
3		32.2	32.1	32.2	−0.2	32.1	31.8	0.8
4		31.2	30.5	31.2	−1.5	30.5	30.0	1.1
Comorbidities (%)								
Hypertension		52.9	48.8	53.2	−8.8	48.8	47.8	2.0
Diabetes mellitus		26.8	26.1	26.8	−1.7	26.1	25.1	2.2
Dyslipidemia		18.6	16.2	18.8	−6.7	16.2	14.9	3.7
Osteoporosis		3.0	24.5	1.7	72.1	24.5	21.2	7.1
Hypoparathyroidism		<0.1	0.2	<0.1	5.0	0.2	<0.1	4.5
HCM		3.4	1.4	3.5	−14.1	1.4	1.6	−2.0
DCM		1.2	1.0	1.3	−2.9	1.0	1.2	−2.8
Cardiac Amyloidosis		0.1	0.1	0.1	−0.8	0.1	0.1	<0.1
Cardiac Sarcoidosis		0.3	0.5	0.3	3.0	0.5	0.2	4.5
Af/AFL		35.5	27.5	36.0	−18.3	27.5	27.2	0.8
AT		0.9	0.6	0.9	−2.9	0.6	0.7	−0.9
Stroke		8.3	7.5	8.3	−3.3	7.5	7.2	1.1
MI		10.3	7.9	10.5	−9.1	7.9	7.2	2.6
PVD		3.8	4.8	3.7	5.1	4.8	3.9	4.3
CKD		14.1	38.6	12.6	62.5	38.6	35.3	6.9
Liver failure		0.1	0.1	0.1	−0.8	0.1	0.2	−2.7
COPD		7.4	6.1	7.5	−5.6	6.1	5.3	3.6
RA		1.3	4.2	1.2	19.0	4.2	4.9	−3.2
Dementia		6.2	6.9	6.2	3.1	6.9	7.0	−0.4
Cancer		11.0	11.0	11.0	0.1	11.0	10.6	1.3
Treatment (%)								
Catecholamine		12.4	11.3	12.5	−3.8	11.3	10.6	2.1
IABP		1.0	0.9	1.0	−1.1	0.9	1.1	−1.7
PCPS		0.1	0.0	0.2	−3.6	0.0	0.1	−0.5
Artificial Ventilation		21.3	21.1	21.3	−0.5	21.1	20.8	0.7
Hemodialysis		4.6	29.5	3.1	76.8	29.5	28.3	2.6
PCI		4.9	5.2	4.9	1.6	5.2	5.1	0.7
Drug (%)								
ACE-i/ARB		19.7	19.4	19.8	−0.8	19.4	21.0	−3.9
βblocker		9.1	8.0	9.2	−4.1	8.0	8.6	−1.9
Loop diuretic		19.4	18.4	19.4	−2.6	18.4	20.3	−4.8
K-sparing diuretic		10.1	7.4	10.3	−10.3	7.4	8.4	−3.7
Statin		13.1	13.5	13.0	1.2	13.5	13.7	−0.7
Hospital length (days)		18 (12–28)	19 (12–31)	17 (12–27)	13.1	19 (12–31)	18 (11–30)	14.9

Data are presented as percentage of patients or median (interquartile range). A standardized difference of < 10% suggests adequate balance. Abbreviations: Vit.D, vitamin D supplementation; std.diff, standardization difference; BMI, body mass index; NYHA, New York heart association functional class; HCM, hypertrophic cardiomyopathy; DCM, dilated cardiomyopathy; Af, atrial fibrillation; AFL, atrial flatter; AT, atrial tachycardia, MI, myocardial infarction; PVD, peripheral vascular disease; CKD, chronic kidney disease; COPD, chronic obstructive pulmonary disease; RA, rheumatoid arthritis, IABP, intra-aortic balloon pumping; PCPS, percutaneous cardiopulmonary system; PCI, percutaneous coronary intervention; ACE-I, angiotensin converting enzyme inhibitor; ARB, angiotensin II receptor blocker.

**Table 2:** In-hospital mortality before and after propensity score matching.

	Non Matching				Matching			
In-Hospital Mortality	Vit.D (+)	Vit.D (−)	OR (95%CI)	*p*-Value	Vit.D (+)	Vit.D (−)	OR (95%CI)	*p*-Value
Total (%)	357 (6.5)	7256 (8.2)	0.79 (0.71–0.88)	<0.0001	357 (6.5)	515 (9.4)	0.67 (0.58–0.77)	<0.0001
7 days (%)	48 (0.9)	1761 (2.0)	0.44 (0.33–0.58)	<0.0001	48 (0.9)	138 (2.5)	0.34 (0.25–0.48)	<0.0001
30 days (%)	207 (3.8)	5171 (5.9)	0.64 (0.55–0.73)	<0.0001	207 (3.8)	358 (6.5)	0.56 (0.47–0.67)	<0.0001

Data given as proportion. Abbreviations: OR, odds ratio.

**Table 3:** Multivariate analysis of covariates for in-hospital mortality.

**A**: In all patients. See abbreviations in Table 1.				
	**All**			
	**OR**	**Lower**	**Higher**	***p***
Vit.D	0.63	0.49	0.81	0.0003
Age	1.06	1.05	1.08	<0.0001
BMI	0.93	0.90	0.96	<0.0001
NYHA	1.18	1.04	1.33	0.0070
Male	1.00	0.74	1.35	0.9884
Smoking	0.93	0.67	1.31	0.6867
HT	0.39	0.30	0.51	<0.0001
DM	0.80	0.60	1.07	0.1398
DL	0.83	0.57	1.22	0.3445
MI	1.17	0.76	1.80	0.4721
PVD	1.88	1.19	2.96	0.0064
Stroke	0.96	0.62	1.50	0.8659
Dementia	1.21	0.78	1.87	0.4000
COPD	0.95	0.56	1.59	0.8370
RA	1.10	0.65	1.89	0.7187
CKD	1.65	1.19	2.30	0.0030
Cancer	0.84	0.57	1.24	0.3764
Hemodialysis	0.84	0.58	1.22	0.3669
Artificial Ventilation	2.55	1.95	3.33	<0.0001
PCI	0.22	0.10	0.47	<0.0001
IABP	1.04	0.44	2.43	0.9355
Catecholamines	4.59	3.50	6.02	<0.0001
Osteoporosis	0.76	0.48	1.19	0.2331
HCM	0.61	0.18	2.09	0.4338
Sarcoidosis	2.60	0.55	12.18	0.2253
Af/AFL	0.68	0.51	0.92	0.0107
**B**: In patients with vitamin D supplementation. See abbreviations in Table 1.				
	**Vitamin D (+)**			
	**OR**	**Lower**	**Higher**	***p***
Age	1.07	1.04	1.09	<0.0001
BMI	0.97	0.92	1.02	0.2186
NYHA	1.22	1.01	1.48	0.0379
Male	0.62	0.37	1.02	0.0589
Smoking	1.10	0.64	1.87	0.7396
HT	0.33	0.22	0.52	<0.0001
DM	0.70	0.44	1.11	0.1257
DL	1.11	0.64	1.90	0.7187
MI	1.36	0.71	2.59	0.3519
PVD	2.24	1.18	4.27	0.0138
Stroke	1.25	0.66	2.37	0.4844
Dementia	1.17	0.59	2.31	0.6536
COPD	0.86	0.38	1.94	0.7122
RA	0.80	0.30	2.12	0.6558
CKD	1.68	0.95	2.99	0.0746
Cancer	1.09	0.60	1.98	0.7663
Hemodialysis	0.98	0.52	1.85	0.9510
Artificial Ventilation	2.18	1.44	3.31	0.0003
PCI	0.19	0.06	0.64	0.0075
IABP	1.34	0.40	4.49	0.6352
Catecholamines	4.91	3.24	7.43	<0.0001
Osteoporosis	0.61	0.36	1.04	0.0707
HCM	1.57	0.34	7.23	0.5659
Sarcoidosis	2.14	0.26	17.31	0.4763
Af/AFL	0.66	0.41	1.06	0.0822
**C**: In patients without vitamin D supplementation. See abbreviations in Table 1.				
	**Vitamin D (−)**			
	**OR**	**Lower**	**Higher**	***p***
Age	1.07	1.04	1.09	<0.0001
BMI	0.91	0.87	0.95	<0.0001
NYHA	1.14	0.97	1.33	0.1111
Male	1.36	0.93	2.01	0.1153
Smoking	0.83	0.54	1.30	0.4209
HT	0.43	0.30	0.60	<0.0001
DM	0.87	0.59	1.28	0.4857
DL	0.64	0.37	1.10	0.1087
MI	1.06	0.59	1.90	0.8573
PVD	1.53	0.79	2.95	0.2038
Stroke	0.77	0.40	1.45	0.4106
Dementia	1.20	0.67	2.15	0.5442
COPD	1.01	0.51	1.99	0.9816
RA	1.19	0.61	2.31	0.6059
CKD	1.55	1.02	2.36	0.0386
Cancer	0.75	0.45	1.26	0.2757
Hemodialysis	0.78	0.48	1.26	0.3036
Artificial Ventilation	3.03	2.12	4.32	<0.0001
PCI	0.25	0.09	0.65	0.0048
IABP	0.74	0.21	2.59	0.6374
Catecholamines	4.72	3.26	6.82	<0.0001
Osteoporosis	1.85	0.76	4.50	0.1746
HCM	0.24	0.03	2.05	0.1939
Sarcoidosis	3.58	0.35	36.60	0.2815
Af/AFL	0.70	0.48	1.02	0.0632

## Data Availability

The datasets are available from the corresponding author on reasonable request.

## Supplementary Materials

The following are available online at https://www.mdpi.com/2072-6643/13/2/335/s1, Figure S1a: Receiver operating characteristic curve and concordance index. Figure S1b: comparison of the consistency of propensity score densities before and after matching. Figure S2: Predictive values for in-hospital mortality.

## References

[1] Tsutsui H., Isobe M., Ito H., Okumura K., Ono M., Kitakaze M., Kinugawa K., Kihara Y., Goto Y., Komuro I. (2019). JCS 2017/JHFS 2017 Guideline on Diagnosis and Treatment of Acute and Chronic Heart Failure- Digest Version. Circ. J..

[2] Heidenreich P.A., Albert N.M., Allen L.A., Bluemke D.A., Butler J., Fonarow G.C., Ikonomidis J.S., Khavjou O., Konstam M.A., Maddox T.M. (2013). Forecasting the impact of heart failure in the United States: A policy statement from the American Heart Association. Circ. Heart Fail..

[3] Wang T.J. (2016). Vitamin D and Cardiovascular Disease. Annu. Rev. Med..

[4] Krul-Poel Y.H., Ter Wee M.M., Lips P., Simsek S. (2017). Management of Endocrine Disease: The effect of vitamin D supplementation on glycaemic control in patients with type 2 diabetes mellitus: A systematic review and meta-analysis. Eur. J. Endocrinol..

[5] Hewison M. (2012). An update on vitamin D and human immunity. Clin. Endocrinol..

[6] Kim D.H., Sabour S., Sagar U.N., Adams S., Whellan D.J. (2008). Prevalence of hypovitaminosis D in cardiovascular diseases (from the National Health and Nutrition Examination Survey 2001 to 2004). Am. J. Cardiol..

[7] Ameri P., Ronco D., Casu M., Denegri A., Bovio M., Menoni S., Ferone D., Murialdo G. (2010). High prevalence of vitamin D deficiency and its association with left ventricular dilation: An echocardiography study in elderly patients with chronic heart failure. Nutr. Metab. Cardiovasc. Dis..

[8] Gardner D.G., Chen S., Glenn D.J. (2013). Vitamin D and the heart. Am. J. Physiol. Regul. Integr. Comp. Physiol..

[9] Schleithoff S.S., Zittermann A., Tenderich G., Berthold H.K., Stehle P., Koerfer R. (2006). Vitamin D supplementation improves cytokine profiles in patients with congestive heart failure: A double-blind, randomized, placebo-controlled trial. Am. J. Clin. Nutr..

[10] Witham M.D., Crighton L.J., Gillespie N.D., Struthers A.D., McMurdo M.E. (2010). The effects of vitamin D supplementation on physical function and quality of life in older patients with heart failure: A randomized controlled trial. Circ. Heart Fail..

[11] Witte K.K., Byrom R., Gierula J., Paton M.F., Jamil H.A., Lowry J.E., Gillott R.G., Barnes S.A., Chumun H., Kearney L.C. (2016). Effects of Vitamin D on Cardiac Function in Patients With Chronic HF: The VINDICATE Study. J. Am. Coll. Cardiol..

[12] Win S., Hussain I., Hebl V.B., Dunlay S.M., Redfield M.M. (2017). Inpatient Mortality Risk Scores and Postdischarge Events in Hospitalized Heart Failure Patients: A Community-Based Study. Circ. Heart Fail..

[13] Peterson P.N., Rumsfeld J.S., Liang L., Albert N.M., Hernandez A.F., Peterson E.D., Fonarow G.C., Masoudi F.A., American Heart Association Get With the Guidelines-Heart Failure Program (2010). A validated risk score for in-hospital mortality in patients with heart failure from the American Heart Association get with the guidelines program. Circ. Cardiovasc. Qual. Outcomes.

[14] Hannan E.L., Wu C., Bennett E.V., Carlson R.E., Culliford A.T., Gold J.P., Higgins R.S., Smith C.R., Jones R.H. (2007). Risk index for predicting in-hospital mortality for cardiac valve surgery. Ann. Thorac. Surg..

[15] Fonarow G.C. (2012). Clinical risk prediction tools in patients hospitalized with heart failure. Rev. Cardiovasc. Med..

[16] Gaksch M., Jorde R., Grimnes G., Joakimsen R., Schirmer H., Wilsgaard T., Mathiesen E.B., Njolstad I., Lochen M.L., Marz W. (2017). Vitamin D and mortality: Individual participant data meta-analysis of standardized 25-hydroxyvitamin D in 26916 individuals from a European consortium. PLoS ONE.

[17] Jiang W.L., Gu H.B., Zhang Y.F., Xia Q.Q., Qi J., Chen J.C. (2016). Vitamin D Supplementation in the Treatment of Chronic Heart Failure: A Meta-analysis of Randomized Controlled Trials. Clin. Cardiol..

[18] Barbarawi M., Kheiri B., Zayed Y., Barbarawi O., Dhillon H., Swaid B., Yelangi A., Sundus S., Bachuwa G., Alkotob M.L. (2019). Vitamin D supplementation and cardiovascular disease risks in more than 83,000 individuals in 21 randomized clinical trials: A meta-analysis. JAMA Cardiol..

[19] Stuart E.A., Bradshaw C.P., Leaf P.J. (2015). Assessing the generalizability of randomized trial results to target populations. Prev. Sci..

[20] Davies N.M., Holmes M.V., Davey Smith G. (2018). Reading Mendelian randomisation studies: A guide, glossary, and checklist for clinicians. BMJ.

[21] Weishaar R.E., Simpson R.U. (1987). Involvement of vitamin D3 with cardiovascular function. II. Direct and indirect effects. Am. J. Physiol..

[22] Chen S., Law C.S., Grigsby C.L., Olsen K., Hong T.T., Zhang Y., Yeghiazarians Y., Gardner D.G. (2011). Cardiomyocyte-specific deletion of the vitamin D receptor gene results in cardiac hypertrophy. Circulation.

[23] Vaidya A., Williams J.S. (2012). The relationship between vitamin D and the renin-angiotensin system in the pathophysiology of hypertension, kidney disease, and diabetes. Metabolism.

[24] Zhang Y., Leung D.Y., Richers B.N., Liu Y., Remigio L.K., Riches D.W., Goleva E. (2012). Vitamin D inhibits monocyte/macrophage proinflammatory cytokine production by targeting MAPK phosphatase-1. J. Immunol..

[25] Caprio M., Mammi C., Rosano G.M. (2012). Vitamin D: A novel player in endothelial function and dysfunction. Arch. Med. Sci..

[26] Schluter K.D., Piper H.M. (1998). Left ventricular hypertrophy and parathyroid hormone: A causal connection?. Cardiovasc. Res..

[27] Haden S.T., Brown E.M., Hurwitz S., Scott J., El-Hajj Fuleihan G. (2000). The effects of age and gender on parathyroid hormone dynamics. Clin. Endocrinol..

[28] Yamana H., Moriwaki M., Horiguchi H., Kodan M., Fushimi K., Yasunaga H. (2017). Validity of diagnoses, procedures, and laboratory data in Japanese administrative data. J. Epidemiol..

