# Supplementary figures and images for: Association between Vitamin D and Heart Failure Mortality in 10,974 Hospitalized Individuals
Source: Nutrients. 2021 Jan 23;13(2):335. doi: 10.3390/nu13020335 (PMC7911510; doi:10.3390/nu13020335)

Supplemental Figure 1:

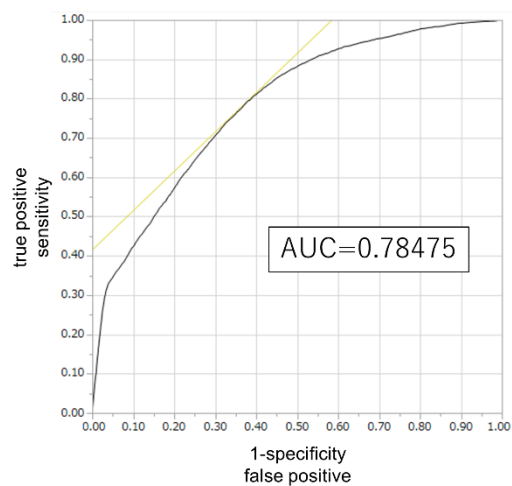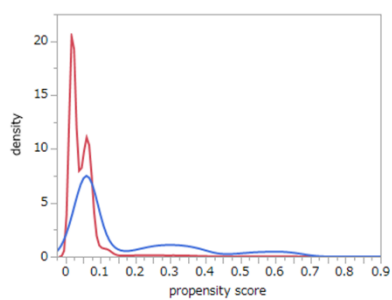

Before matching

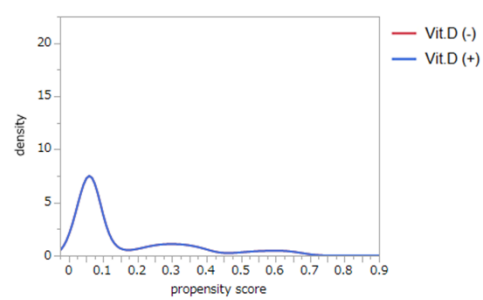

After matching

Supplemental Figure 2:

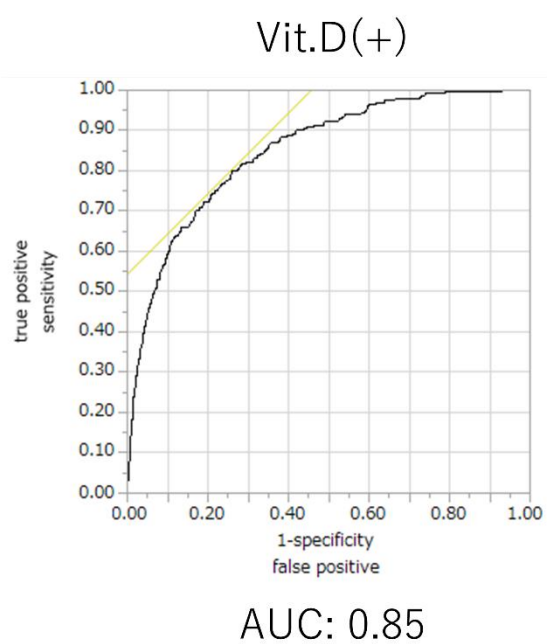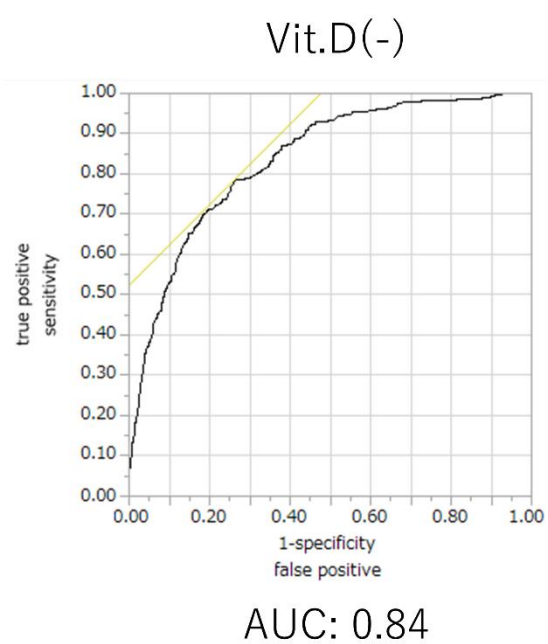

Supplement: Supplementary file 1 [file nutrients-13-00335-s001.pdf]
